# Dual-Energy CT for the Detection of Portal Vein Thrombosis: Improved Diagnostic Performance Using Virtual Monoenergetic Reconstructions

**DOI:** 10.3390/diagnostics12071682

**Published:** 2022-07-10

**Authors:** Simon S. Martin, Jetlir Kolaneci, Rouben Czwikla, Christian Booz, Leon D. Gruenewald, Moritz H. Albrecht, Zachary M. Thompson, Lukas Lenga, Ibrahim Yel, Thomas J. Vogl, Julian L. Wichmann, Vitali Koch

**Affiliations:** 1 Department of Diagnostic and Interventional Radiology, University Hospital Frankfurt, 60590 Frankfurt, Germany; jetlir@gmail.com (J.K.); rouben.czwikla@gmail.com (R.C.); christian.booz@kgu.de (C.B.); leondavid.gruenewald@kgu.de (L.D.G.); moritzalbrecht@gmx.net (M.H.A.); lukas.lenga@kgu.de (L.L.); ibrahim.yel@kgu.de (I.Y.); thomas.vogl@kgu.de (T.J.V.); docwichmann@gmail.com (J.L.W.); vitali.koch@kgu.de (V.K.); 2 Department of Radiology and Radiological Science, Medical University of South Carolina, Charleston, SC 29425, USA; thompsoz@musc.edu

**Keywords:** diagnostic imaging, liver, multidetector computed tomography, portal vein, thrombosis

## Abstract

**Purpose:** To investigate the diagnostic performance of noise-optimized virtual monoenergetic images (VMI+) in dual-energy CT (DECT) of portal vein thrombosis (PVT) compared to standard reconstructions. **Method:** This retrospective, single-center study included 107 patients (68 men; mean age, 60.1 ± 10.7 years) with malignant or cirrhotic liver disease and suspected PVT who had undergone contrast-enhanced portal-phase DECT of the abdomen. Linearly blended (M_0.6) and virtual monoenergetic images were calculated using both standard VMI and noise-optimized VMI+ algorithms in 20 keV increments from 40 to 100 keV. Quantitative measurements were performed in the portal vein for objective contrast-to-noise ratio (CNR) calculation. The image series showing the greatest CNR were further assessed for subjective image quality and diagnostic accuracy of PVT detection by two blinded radiologists. **Results:** PVT was present in 38 subjects. VMI+ reconstructions at 40 keV revealed the best objective image quality (CNR, 9.6 ± 4.3) compared to all other image reconstructions (*p* < 0.01). In the standard VMI series, CNR peaked at 60 keV (CNR, 4.7 ± 2.1). Qualitative image parameters showed the highest image quality rating scores for the 60 keV VMI+ series (median, 4) (*p* ≤ 0.03). The greatest diagnostic accuracy for the diagnosis of PVT was found for the 40 keV VMI+ series (sensitivity, 96%; specificity, 96%) compared to M_0.6 images (sensitivity, 87%; specificity, 92%), 60 keV VMI (sensitivity, 87%; specificity, 97%), and 60 keV VMI+ reconstructions (sensitivity, 92%; specificity, 97%) (*p* ≤ 0.01). **Conclusions:** Low-keV VMI+ reconstructions resulted in significantly improved diagnostic performance for the detection of PVT compared to other DECT reconstruction algorithms.

## 1. Introduction

Portal vein thrombosis (PVT) is a frequently encountered complication in patients with hepatic malignancy or liver cirrhosis [1,2]. The early detection of PVT is essential in these subjects to provide therapeutic intervention and prevent irreversible organ damage [2,3]. The detection or rule-out of PVT can be challenging because decreased blood flow in the portal and intrahepatic vessels in patients with liver diseases often leads to poor intravascular contrast opacification [3].

Several studies have demonstrated that virtual monoenergetic imaging (VMI) in dual-energy CT (DECT) allows for the optimization of contrast conditions and image quality [4,5,6,7]. As iodine attenuation increases with decreasing kilo electron volt (keV) levels towards the iodine k-edge at 33 keV, vascular enhancement can be improved using VMI DECT reconstructed at low energies. However, this option was limited to using traditional VMI reconstructions at the very low-keV range because of a concurrent increase in image noise [4,6,7,8]. A refined image-based algorithm (VMI+), however, has been shown to overcome this limitation and provide a superior contrast-to-noise ratio (CNR) at low energies (40–60 keV) compared to both its predecessor technique and standard linearly blended DECT image reconstruction [9,10,11]. In a prior study, Schabel et al. found superior CNR and subjective image quality for the visualization of intrahepatic vessels at 40 keV [12]. Furthermore, Bongers et al. reported on the improved delineation of thrombotic material in a phantom model using VMI+ [13]. However, the actual impact of DECT-derived monoenergetic reconstructions on the diagnostic accuracy for the detection of PVT in vivo has not been investigated to date.

Therefore, we aimed to investigate the impact of the noise-optimized VMI+ reconstruction algorithm on the visualization, diagnostic confidence, and accuracy for the detection of PVT in patients with liver disease.

## 2. Materials and Methods

### 2.1. Study Cohort

This single-center study was approved by the local institutional review board with a waiver for written informed consent. We retrospectively analyzed our institutional CT databases to identify patients with malignant or cirrhotic liver disease and suspected PVT who had undergone DECT examinations between September 2014 and October 2017 within the clinical routine. The query yielded an initial target population of 174 subjects. Patients without an adequate reference standard (*n* = 51), as well as patients with severe motion artifacts (*n* = 4) or deviations from the standard contrast media injection protocol (*n* = 12), were excluded. For an estimation of the CT radiation dose, the volume CT dose index (CDTI_vol_), the dose length product (DLP), and the effective dose of each examination were recorded.

**Figure 1:**
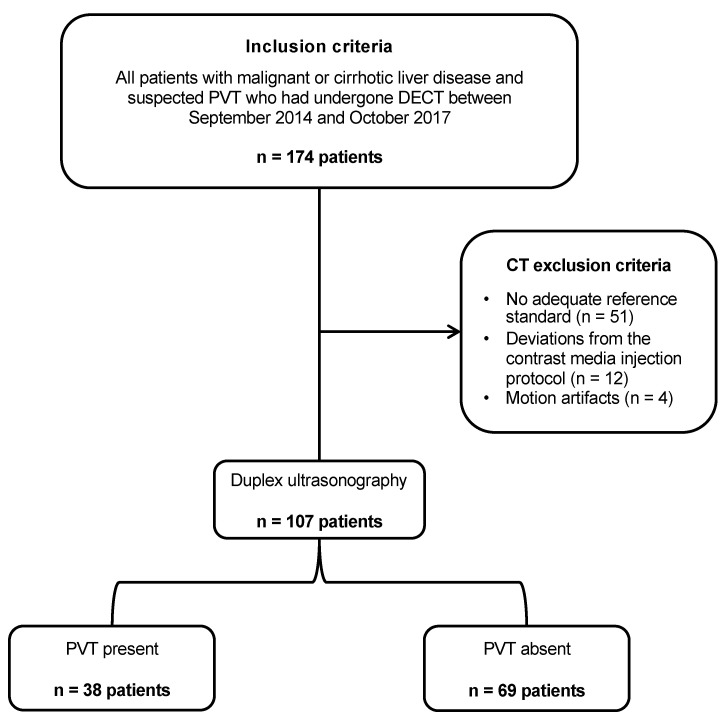
The analytic population included 107 patients with malignant or cirrhotic liver disease and suspected portal vein thrombosis (PVT) who had undergone dual-energy CT (DECT) examinations of the abdomen. PVT = portal vein thrombosis.

The final study population consisted of 107 patients (mean age, 60.1 ± 10.7 years), including 68 men (mean age, 59.9 ± 9.2 years) and 39 women (mean age, 60.4 ± 12.8 years) (Table 1). The mean body mass index was 25.1 ± 6.7 kg/m^2^. Figure 1 presents a flowchart of patient inclusion based on recommended Standards for Reporting of Diagnostic Accuracy criteria (STARD) [14].

### 2.2. Reference Standard

In total, 38 (36%) of the 107 patients recruited in our study were diagnosed with PVT. The entity was considered present on contrast-enhanced DECT when a hypodense-filling defect within the lumen of the portal vein (partial occlusion) or no portal vein opacification (complete occlusion) could be observed. In the group of patients with PVT, a total of 12 patients showed an extension of the thrombus into portal vein branches (*n* = 5), the splenic vein (*n* = 2), or the superior mesenteric vein (*n* = 5). Duplex ultrasonography was performed within two weeks before or after the DECT (median of 5 days, range 1–14 days) in all subjects, confirming the presence or absence of PVT. An additional consensus reading was performed for all patients by two radiologists with more than 8 years of experience in abdominal CT imaging (J.L.W. and L.L.) to determine the final diagnosis based on all available clinical and imaging data. No conflicts were reported between the radiologic interpretation of the DECT and sonography data.

### 2.3. DECT Technique

DECT examinations were performed using a third-generation dual-source DECT scanner (Somatom Force, Siemens Healthineers, Forchheim, Germany) operated in dual-energy mode. The two X-ray tubes were set to different kV tube voltages (tube A, 80–100 kV and tube B, 150 kV with tin filter). The reference tube current–time products for tube A and tube B were 190 mAs and 95 mAs, respectively. Furthermore, the following settings were used: rotation time, 0.5 s; collimation, 2 × 192 × 0.6 mm; and pitch, 0.6. Patients were scanned in expiratory breath hold from the anterior diaphragm to the aortic bifurcation. Non-ionic contrast media (Imeron 350, Bracco, Milan, Italy) at a dose of 1.2 mL per kilogram of body weight with a maximum of 120 mL were injected at a flow rate of 3 mL/s through a peripheral vein of the forearm, followed by an 80 mL saline flush. The arterial phase automatically started 7 s after a threshold of 120 Hounsfield units (HU) was reached in the descending aorta using bolus tracking software (CareBolus, Siemens Healthineers). Portal-venous DECT imaging data were acquired 80 s after contrast media injection. Only portal-venous phase DECT data were used for further post-processing and image analysis.

Standard M_0.6 linearly blended images were reconstructed, merging 60% of the low-keV and 40% of the high-keV spectra and using third-generation Advanced Modeled Iterative Reconstruction software (Admire, Siemens Healthineers; strength level, 3) with a smooth tissue kernel (Qr36). Furthermore, monoenergetic images were reconstructed at energy levels ranging from 40–100 keV in 20 keV increments using the VMI and VMI+ technique, respectively, on a commercially available 3D Workstation (syngo.via. version VB10B, Siemens Healthineers). All series were reconstructed with a section thickness of 3 mm and an increment of 2 mm.

### 2.4. Quantitative Analysis

A radiologist with 3 years of experience in abdominal CT imaging (I.Y.) measured attenuation differences (given in HU) in the portal vein and portal branch (prior and subsequent to the thrombus formation, if visible), as well as in the splenic and superior mesenteric vein. Furthermore, attenuation measurements were performed in the psoas major muscle and abdominal fat. For each measurement, the region of interest (ROI) was drawn as large as possible, while the inclusion of surrounding perivascular tissue was carefully avoided. The standard deviation (SD) for each attenuation value was recorded. In accordance with prior studies, we calculated the signal-to-noise (SNR) and contrast-to-noise ratios (CNR) using the following formulas [4,10,11]:

$$\mathrm{SNR}=\frac{\mathrm{mean}\;\mathrm{signal}\;\mathrm{intensity}\;(\mathrm{portal}\;\mathrm{vein})}{\mathrm{standard}\;\mathrm{deviation}\;\mathrm{of}\;\mathrm{attenuation}\;(\mathrm{subcutaneous}\;\mathrm{fat})}$$

$$\mathrm{CNR}=\frac{\mathrm{mean}\;\mathrm{signal}\;\mathrm{intensity}\;\left(\mathrm{portal}\;\mathrm{vein}\right)\;-\;\mathrm{mean}\;\mathrm{signal}\;\mathrm{intensity}\;(\mathrm{psoas}\;\mathrm{muscle})}{\mathrm{standard}\;\mathrm{deviation}\;\mathrm{of}\;\mathrm{attenuation}\;(\mathrm{subcutaneous}\;\mathrm{fat})}$$

### 2.5. Qualitative Analysis

Two radiologists with 4 and 5 years of experience in CT imaging (M.H.A. and S.S.M.) participated in the qualitative image analysis, which was performed in several reading sessions for M_0.6, 60 keV VMI, 40 keV VMI+, and 60 keV VMI+ series. The decision to assess monoenergetic images at the three aforementioned keV values was based on the quantitative image analysis and previous studies that have reported the highest subjective image quality at 60 keV [6,15]. One series per patient was evaluated during each read-out. A time interval of at least one week was kept between ratings to minimize potential recall bias. For all criteria, five-point Likert scales were used. Noise was assessed (ranging from 1 = extensive noise to 5 = sharp image without perceivable noise), in addition to contrast (ranging from 1 = poor contrast to 5 = excellent contrast), subjective image quality (1 = poor image quality to 5 = excellent image quality), and suitability for PVT assessment (ranging from 1 = inappropriate for PVT assessment to 5 = outstanding suitability to assess PVT).

### 2.6. Diagnostic Accuracy

Diagnostic accuracy analyses were performed for the same image reconstructions that were assessed in the qualitative image analysis (M_0.6, 60 keV VMI, 40 keV VMI+, and 60 keV VMI+). DECT images were independently assessed by the same two radiologists who participated in the qualitative image analysis. Reviewers were asked to score their diagnostic confidence regarding the presence or absence of PVT (ranging from 1 = no evidence of PVT, to 5 = PVT is certainly present) [11]. The radiologists were allowed to freely alter window settings to adjust contrast and signal intensity. Readers were aware that the study focused on the imaging and detection of PVT but were blinded to all clinical findings and the number of included patients that were negative for PVT.

### 2.7. Statistical Analysis

Statistical analysis was performed using commercially available software (MedCalc Statistical Software for Windows, Version 17.9, Ostend, Belgium). The Kolmogorov–Smirnov test was used to examine the normality of data distribution. Continuous variables are expressed as means ± standard deviation (SD), and ordinal variables are expressed as medians with interquartile ranges (IQR). Categorical variables are presented as numbers with corresponding percentages. Subsequently, data showing a Gaussian distribution were evaluated using the analysis of variance (ANOVA). For data of unequal variance, the Wilcoxon matched-pairs test was applied.

Interobserver agreement among the two reviewers was assessed by means of Cohen’s kappa coefficient (κ) analysis and interpreted as follows [16]: κ = <0.20, poor agreement, κ = 0.21–0.40, fair agreement, κ = 0.41–0.60, moderate agreement, κ = 0.61–0.80, good agreement, and κ = 0.81–1.0, excellent agreement.

In an intention-to-diagnose approach, PVT detection confidence scores of 1 and 2 were regarded as negative, and scores of 3–5 were considered positive for PVT and compared to the reference standard [17,18]. Receiver-operating characteristic (ROC) analysis with area under the curve (AUC) values was calculated. Accuracy, sensitivity, and specificity were determined from the defined threshold by the Youden index. For all used tests, a *p* value ≤ 0.05 was considered to indicate a statistically significant result.

## 3. Results

The mean CDTI_vol_ of all examinations was 7.7 ± 1.8 mGy, and the mean DLP was 396.9 ± 2.9 mGy·cm. This resulted in an average effective dose of 6.7 ± 2.9 mSv.

### 3.1. Quantitative Analysis

**Figure 2:**
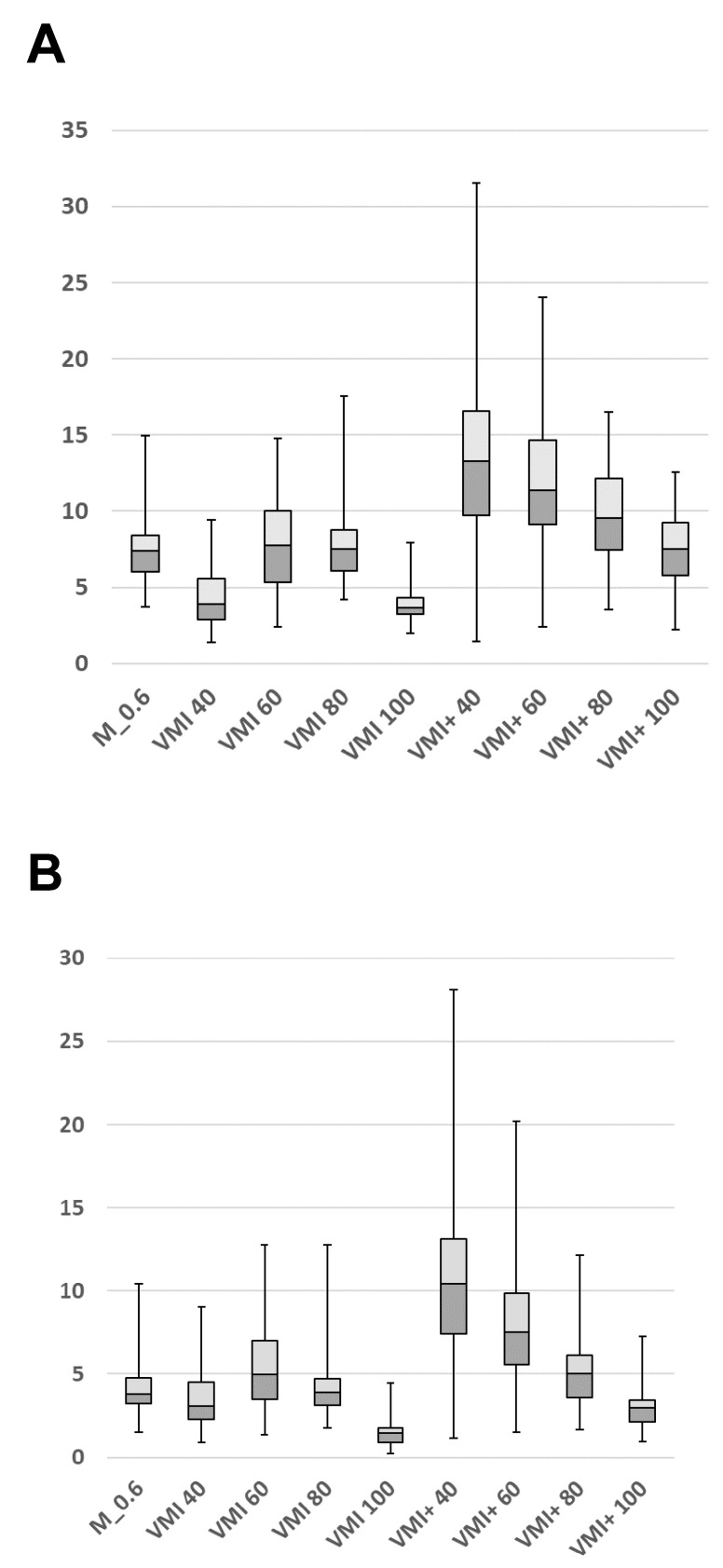
The box-and-whisker plots demonstrate the results of the quantitative image analysis (variables are presented as means ± SD). Signal-to-noise (SNR, **A**) and contrast-to-noise ratios (CNR, **B**) in the portal vein were compared between linearly blended M_0.6, standard virtual monoenergetic imaging (VMI), and noise-optimized VMI+ series at different keV levels.

**Figure 3:**
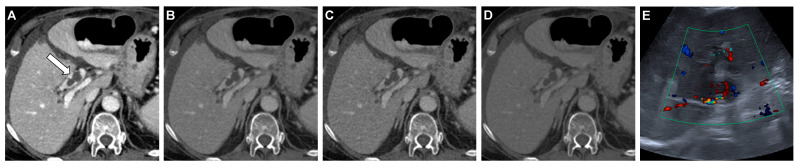
Axial DECT images of a 90-year-old woman with liver cirrhosis and portal vein thrombosis (*arrow*). Noise-optimized virtual monoenergetic imaging (VMI+) series at 40 keV (**A**) show higher vascular attenuation of the portal vein and a better delineation of the thrombus compared to 60 keV VMI+ (**B**), 60 keV VMI (**C**), and standard linearly blended M_0.6 images (**D**). On color duplex ultrasonography, some color signals are visible within the boundaries of the thrombus (**E**).

Quantitative image quality parameters are summarized in Table 2 and illustrated in Figure 2. The highest intravenous attenuation was found for the 40 keV VMI and 40 keV VMI+ series (396.3 ± 106.4 and 390.7 ± 59.4 HU, respectively), without significant differences (*p* > 0.05). However, significantly higher noise was found for traditional VMI images at 40–60 keV compared to all other reconstructions (*p* < 0.01). The quantitative image quality of VMI+ revealed a gradual increase from high to low keV levels, showing the greatest CNR at 40 keV (9.6 ± 4.3), which was significantly superior compared to all other series (*p* < 0.01). VMI+ series at 60 keV also showed superior quantitative image quality in comparison with M_0.6 images (CNR, 7.0 ± 2.8 vs. 3.8 ± 1.7) and all traditional VMI series (*p* < 0.01). The 60 keV VMI reconstructions showed the best objective image quality of the traditional VMI series (CNR, 4.7 ± 2.1), which was also significantly increased compared to the M_0.6 series (*p* < 0.01) (Figure 3). VMI reconstructions at 80 and 100 keV differed not significantly from the corresponding VMI+ series (*p* = 0.30 and *p* = 0.64, respectively).

### 3.2. Qualitative Analysis

**Figure 4:**
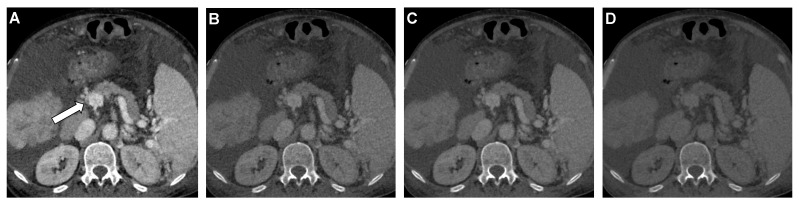
Coronal DECT images of a 67-year-old man with subtle portal vein thrombosis at the confluence of the superior mesenteric and splenic vein (*arrow*). The thrombus is best visible in 40 keV noise-optimized virtual monoenergetic imaging (VMI+) (**A**), followed by the 60 keV VMI+ series (**B**), whereas the detection using 60 keV VMI (**C**) and standard M_0.6 (**D**) series is hindered.

The ratings of the qualitative image analysis are summarized in Table 3, including the associated κ values. Intravenous contrast was rated superior for VMI+ reconstructions at 40 keV (*p* < 0.01) with moderate agreement among readers (κ, 0.58). Noise ratings for 40 keV VMI+ and 60 keV VMI series (medians, 2 and 3, respectively) were significantly lower than those of M_0.6 and 60 keV VMI+ reconstructions (both medians, 4) (*p* < 0.01). VMI+ images at 60 keV were rated the best regarding subjective image quality (median, 4) (*p* ≤ 0.03; κ, 0.58), while VMI+ images at 40 keV showed the highest suitability for PVT assessment (median, 4) compared to the three other evaluated series (*p* < 0.01) (Figure 4) and revealed a good agreement between both raters (κ, 0.63).

### 3.3. Diagnostic Accuracy

**Figure 5:**
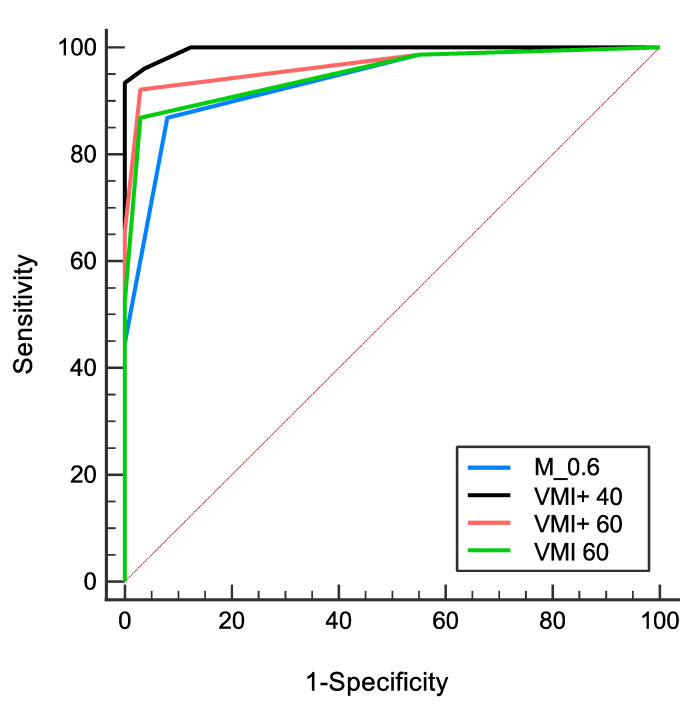
Results of the diagnostic performance analysis. The receiver operating characteristic (ROC) curves, derived from the ratings of the two readers for the detection of portal vein thrombosis, were highest for 40 keV noise-optimized virtual monoenergetic imaging (VMI+) series (AUC, 0.99) compared to 60 keV VMI+ (AUC, 0.97), 60 keV VMI (AUC, 0.95), and M_0.6 image reconstructions (AUC, 0.94) (*p* < 0.01).

The diagnostic performance for the diagnosis of PVT was highest using 40 keV VMI+ series (sensitivity, 96%; specificity, 96%; AUC, 0.99) compared to all other reconstructions (all *p* ≤ 0.01). Furthermore, VMI+ at 60 keV (sensitivity, 92%; specificity, 97%; AUC, 0.97) showed a superior diagnostic performance compared to 60 keV VMI (sensitivity, 87%; specificity, 97%; AUC, 0.95) and M_0.6 image reconstructions (sensitivity, 87%; specificity, 92%; AUC, 0.94). Table 4 summarizes the indices of diagnostic performance. Figure 5 illustrates the AUC of the ROC analyses.

## 4. Discussion

This study aimed to assess the impact of the VMI+ algorithm on the visualization and diagnosis of PVT in patients with malignant or cirrhotic liver disease. We found enhanced diagnostic accuracy for the detection of PVT using 40 keV VMI+ reconstructions. Furthermore, our data show that low-keV VMI+ images were found most suitable by the radiologists for analyzing PVT and provided the greatest diagnostic confidence compared to traditional VMI and standard image series.

The noise-optimized VMI+ algorithm has been specifically developed to enable a high image contrast without an increase in noise, which is known to be a major limitation of traditional VMI [5]. In a previous study, Schabel et al. demonstrated image quality improvements using VMI+ reconstructions at 40 keV compared to VMI and linear-blended images in 25 patients with poor contrast conditions of the liver [12]. Our results are consistent with these findings, as 40 keV series revealed the highest CNR. More importantly, our data indicate that this increase in venous contrast opacification translates into greater diagnostic accuracy for PVT detection. Such improved diagnostic performance for thrombus assessment has been previously assumed by Bongers et al., who demonstrated the benefits of VMI+ series at 40 keV for thrombus delineation in a phantom model [13]. We therefore recommend that VMI+ should be preferred over VMI in DECT of hepatic and mesenteric veins. Furthermore, other investigators demonstrated that low-keV VMI+ results in improved diagnostic performance for the diagnosis of pulmonary embolism in poor contrast conditions or the scenario when only a venous-phase acquisition is available [19,20]. Both aforementioned in vivo studies and our current investigations are in accordance using the same principle of enhancing vascular iodine attenuation for improved detection of thrombotic material.

Notably, observers preferred VMI+ reconstructions at 60 keV in the qualitative image analysis of our study. We presume that a substantial increase in contrast cannot completely outweigh the noise increase in 40 keV VMI+ reconstructions in terms of general subjective image quality, even though providing higher CNR and ultimately enhanced detection rate of PVT. This explanation has also been suggested in other recent studies investigating VMI+ [9,11,21]. 

The results of our study could potentially have important clinical implications. Suboptimal contrast opacification of the portal vein in CT examinations remains a limiting factor in both the diagnostic confidence and accuracy of PVT assessment [22]. In patients with chronic liver disease, portal hypertension usually leads to a decreased inflow of contrast agent into the portal vein from the splanchnic circulation. Increasing the amount of contrast material a priori is mostly not a suitable solution, as patients with liver disease suffer frequently from concomitant renal impairment (for instance, in the scenario of hepato-renal syndrome) [23,24]. Even though the debate regarding the influence of intravenous administration of iodinated contrast material on renal function is ongoing [25,26,27], limiting the amount of contrast media remains a priority to avoid contrast-induced nephropathy [28,29,30]. In this context, the reconstruction of low-keV noise-optimized VMI+ series may be a viable solution for dose-neutral enhanced contrast opacification of the portal vein and better delineation of potential intraluminal thrombus. The feasibility of this approach may further be supported by the recent innovation that low-keV VMI+ images can be reconstructed automatically and routinely from the DECT scanner console without requiring mandatory manual reconstruction.

Several limitations of our study need to be considered. First, we enrolled patients retrospectively and our study population was composed entirely of patients with chronic or malignant liver disease. The absence of other diseases may limit the applicability of our results to other types of PVT. Second, since we reconstructed nine image series in total for the quantitative image analysis, we only evaluated VMI and VMI+ series in 20 keV intervals from 40 to 100 keV and omitted the analysis of energy levels of 50, 70, and 90 keV. The results may vary using different keV levels. Third, we stipulated rating scores of 1–2 as negative and scores of 3–5 as positive for the absence or presence of PVT, respectively. This might have influenced the diagnostic performance analysis and artificially increased the observed sensitivity, although the intention-to-treat approach is commonly used for such diagnostic accuracy studies [17,18]. Finally, we assessed only the portal venous phase of contrast enhancement in this study without consideration of unenhanced and arterial phase images. However, unenhanced scans have shown only minimal benefits for thrombus detection [31].

In conclusion, we demonstrated that low-keV VMI+ reconstructions at 40 keV allow for significantly improved diagnostic performance for the detection of PVT compared to other DECT reconstruction algorithms.

## Figures and Tables

**Table 1:** Patient characteristics.

Characteristic	Value
Age (years)	60.1 ± 10.7
Male patients	68 (64%)
Female patients	39 (36%)
BMI (kg/m^2^)	25.1 ± 6.7
PVT present	38 (36%)
PVT absent	69 (64%)
Liver disease:	
Liver cirrhosis	*n* = 27
HCC	*n* = 35
Liver metastasis	*n* = 45

Age (years) and BMI (kg/m^2^) are presented as mean value ± standard deviation (SD). Other data are numbers of patients, with percentages in parenthesis. BMI = body mass index. PVT = portal vein thrombosis. HCC = hepatocellular carcinoma.

**Table 2:** Quantitative image analysis. Portal vein attenuation (HU), signal-to-noise (SNR) and contrast-to-noise ratio (CNR) were compared between linearly blended M_0.6, standard virtual monoenergetic imaging (VMI), and noise-optimized VMI+ series at different keV-levels in various selected abdominal areas.

	M_0.6	40 VMI	60 VMI	80 VMI	100 VMI	40 VMI+	60 VMI+	80VMI+	100 VMI+
**Portal vein** **attenutation (HU)**	127.9 ± 23.8	396.3 ± 106.4	195.5 ± 44.0	117.9 ± 22.6	84.8 ± 32.3	390.7 ± 59.4	194.7 ± 29.7	119.5 ± 18.7	86.1 ± 16.6
**SNR**									
Portal vein	7.5 ± 2.0	4.2 ± 1.7	7.7 ± 3.1	7.6 ± 2.2	3.8 ± 1.0	14.0 ± 5.7	11.7 ± 4.1	9.7 ± 3.1	7.6 ± 2.3
Portal branch	7.5 ± 2.1	4.2 ± 1.7	7.6 ± 2.9	7.7 ± 2.1	3.8 ± 1.0	13.1 ± 5.1	11.3 ± 3.6	9.8 ± 2.8	7.9 ± 2.2
SMV	7.2 ± 2.0	4.2 ± 1.7	7.7 ± 3.2	7.6 ± 2.3	3.7 ± 1.1	14.0 ± 5.9	11.5 ± 4.1	9.5 ± 3.0	7.3 ± 2.1
Splenic vein	7.7 ± 2.5	4.4 ± 1.7	8.0 ± 3.2	7.9 ± 2.4	3.9 ± 1.1	14.7 ± 5.9	12.2 ± 4.1	10.1 ± 3.1	7.8 ± 2.3
Liver parenchyma	5.4 ± 1.4	2.2 ± 0.9	4.8 ± 0.9	5.8 ± 1.5	3.3 ± 0.8	7.4 ± 3.1	7.3 ± 2.5	7.3 ± 2.2	6.6 ± 1.9
***Average***	7.1 ± 2.0	3.8 ± 1.5	7.1 ± 2.9	7.3 ± 2.1	3.7 ± 1.0	12.6 ± 5.1	10.8 ± 3.7	9.3 ± 2.8	7.5 ± 2.1
**CNR**									
Portal vein	4.2 ± 1.6	3.4 ± 1.5	5.3 ± 2.3	4.1 ± 1.6	1.4 ± 0.7	11.0 ± 4.8	7.9 ± 3.2	5.1 ± 2.1	2.9 ± 1.4
Portal branch	4.2 ± 1.8	3.4 ± 1.5	5.1 ± 2.2	4.1 ± 1.7	1.4 ± 0.9	10.1 ± 4.4	7.5 ± 2.9	5.2 ± 2.0	3.2 ± 1.5
SMV	3.9 ± 1.7	3.4 ± 1.5	5.2 ± 2.4	4.0 ± 1.8	1.3 ± 0.8	11.0 ± 5.0	7.7 ± 3.2	4.9 ± 2.0	2.6 ± 1.3
Splenic vein	4.4 ± 2.1	3.6 ± 1.6	5.5 ± 2.5	4.3 ± 1.9	1.5 ± 0.8	11.7 ± 5.0	8.3 ± 3.3	5.5 ± 2.3	3.1 ± 1.6
Liver parenchyma	2.1 ± 1.0	1.4 ± 0.7	2.4 ± 1.1	2.2 ± 0.9	0.9 ± 0.5	4.4 ± 2.3	3.5 ± 1.6	2.6 ± 1.2	1.9 ± 1.0
***Average***	3.8 ± 1.7	3.0 ± 1.4	4.7 ± 2.1	3.7 ± 1.6	1.3 ± 0.7	9.6 ± 4.3	7.0 ± 2.8	4.7 ± 1.9	2.7 ± 1.4

Data are means ± standard deviation. VMI = virtual monoenergetic imaging, VMI+ = noise-optimized virtual monoenergetic imaging, HU = Hounsfield unit, SNR = signal-to-noise ratio, CNR = contrast-to-noise ratio, SMV = superior mesenteric vein.

**Table 3:** Qualitative image analysis. The image parameters image noise, image contrast, image quality, and suitability were compared between linearly blended M_0.6, standard virtual monoenergetic imaging (VMI) at 60 keV, and noise-optimized VMI+ series at 40 keV and 60 keV levels.

Parameter	M_0.6	60 keV VMI	40 keV VMI+	60 keV VMI+
Image noise	4 (2–5) [0.62; 0.49–0.76]	3 (1–4) [0.63; 0.50–0.76]	2 (1–4) [0.63; 0.50–0.76]	4 (4–5) [0.64; 0.49–0.79]
Image contrast	3 (2–4) [0.25; 0.07–0.44]	3 (1–4) [0.54; 0.36–0.71]	5 (3–5) [0.58; 0.43–0.73]	4 (2–5) [0.47; 0.31–0.63]
Image quality	3 (2–4) [0.54; 0.39–0.69]	3 (1–4) [0.67; 0.54–0.81]	3 (1–4) [0.46; 0.30–0.63]	4 (3–5) [0.58; 0.42–0.73]
Suitability	3 (1–4) [0.61; 0.47–0.76]	3 (1–4) [0.33; 0.17–0.50]	4 (3–5) [0.63; 0.49–0.77]	3 (2–5) [0.58; 0.44–0.72]

Results of the qualitative image analysis are reported as medians with interquartile ranges in parenthesis. Cohen’s kappa coefficients (κ) with 95% confidence intervals (CI) are displayed in brackets. VMI = virtual monoenergetic imaging, VMI+ = noise-optimized virtual monoenergetic imaging.

**Table 4:** Diagnostic performance of linearly blended M_0.6, standard virtual monoenergetic imaging (VMI) at 60 keV, and noise-optimized VMI+ series at 40 keV and 60 keV levels for the detection of portal vein thrombosis (PVT).

Parameter	M_0.6	60 keV VMI	40 keV VMI+	60 keV VMI+
Sensitivity	87% [0.77–0.94]	87% [0.77–0.94]	96% [0.89–0.99]	92% [0.84–0.97]
Specificity	92% [0.87–0.96]	97% [0.93–0.99]	96% [0.93–0.99]	97% [0.93–0.99]
AUC	0.94 [0.90–0.97]	0.95 [0.91–0.98]	0.99 [0.98–1.00]	0.97 [0.93–0.99]
*p*-value	*p* < 0.001	*p* = 0.001	N/A	*p* = 0.012

Sensitivity and specificity are presented as percentages with corresponding 95% confidence intervals (CI) in brackets. VMI = virtual monoenergetic imaging, VMI+ = noise-optimized virtual monoenergetic imaging, AUC = Area under the curve. *p*-values refer to the comparison with 40 keV VMI+ values.

## Data Availability

Data are available on a reasonable request from the authors.

## Abbreviations

CNR	Contrast-to-noise ratio
DECT	Dual-energy computed tomography
PVT	Portal vein thrombosis
VMI	Virtual monoenergetic images
VMI+	Noise-optimized virtual monoenergetic images

## References

[1] Janssen H.L., Wijnhoud A., Haagsma E.B., van Uum S.H., van Nieuwkerk C.M., Adang R.P., Chamuleau R.A., van Hattum J., Vleggaar F.P., Hansen B.E. (2001). Extrahepatic portal vein thrombosis: Aetiology and determinants of survival. Gut.

[2] Sogaard K.K., Astrup L.B., Vilstrup H., Gronbaek H. (2007). Portal vein thrombosis; risk factors, clinical presentation and treatment. BMC Gastroenterol..

[3] Hidajat N., Stobbe H., Griesshaber V., Felix R., Schroder R.J. (2005). Imaging and radiological interventions of portal vein thrombosis. Acta Radiol..

[4] Apfaltrer P., Sudarski S., Schneider D., Nance J.W., Haubenreisser H., Fink C., Schoenberg S.O., Henzler T. (2014). Value of monoenergetic low-kV dual energy CT datasets for improved image quality of CT pulmonary angiography. Eur. J. Radiol..

[5] Grant K.L., Flohr T.G., Krauss B., Sedlmair M., Thomas C., Schmidt B. (2014). Assessment of an advanced image-based technique to calculate virtual monoenergetic computed tomographic images from a dual-energy examination to improve contrast-to-noise ratio in examinations using iodinated contrast media. Investig. Radiol..

[6] Schneider D., Apfaltrer P., Sudarski S., Nance J.W., Haubenreisser H., Fink C., Schoenberg S.O., Henzler T. (2014). Optimization of kiloelectron volt settings in cerebral and cervical dual-energy CT angiography determined with virtual monoenergetic imaging. Acad. Radiol..

[7] Sudarski S., Apfaltrer P., Nance J.W., Schneider D., Meyer M., Schoenberg S.O., Fink C., Henzler T. (2013). Optimization of keV-settings in abdominal and lower extremity dual-source dual-energy CT angiography determined with virtual monoenergetic imaging. Eur. J. Radiol..

[8] Husarik D.B., Gordic S., Desbiolles L., Krauss B., Leschka S., Wildermuth S., Alkadhi H. (2015). Advanced virtual monoenergetic computed tomography of hyperattenuating and hypoattenuating liver lesions: Ex-vivo and patient experience in various body sizes. Investig. Radiol..

[9] Albrecht M.H., Scholtz J.E., Husers K., Beeres M., Bucher A.M., Kaup M., Martin S.S., Fischer S., Bodelle B., Bauer R.W. (2016). Advanced image-based virtual monoenergetic dual-energy CT angiography of the abdomen: Optimization of kiloelectron volt settings to improve image contrast. Eur. Radiol..

[10] Martin S.S., Wichmann J.L., Weyer H., Albrecht M.H., D’Angelo T., Leithner D., Lenga L., Booz C., Scholtz J.E., Bodelle B. (2017). Dual-energy computed tomography in patients with cutaneous malignant melanoma: Comparison of noise-optimized and traditional virtual monoenergetic imaging. Eur. J. Radiol..

[11] Martin S.S., Wichmann J.L., Scholtz J.E., Leithner D., D’Angelo T., Weyer H., Booz C., Lenga L., Vogl T.J., Albrecht M.H. (2017). Noise-Optimized Virtual Monoenergetic Dual-Energy CT Improves Diagnostic Accuracy for the Detection of Active Arterial Bleeding of the Abdomen. J. Vasc. Interv. Radiol..

[12] Schabel C., Bongers M., Sedlmair M., Korn A., Grosse U., Mangold S., Claussen C.D., Thomas C. (2014). Assessment of the hepatic veins in poor contrast conditions using dual energy CT: Evaluation of a novel monoenergetic extrapolation software algorithm. RöFo.

[13] Bongers M.N., Schabel C., Krauss B., Tsiflikas I., Ketelsen D., Mangold S., Claussen C.D., Nikolaou K., Thomas C. (2015). Noise-optimized virtual monoenergetic images and iodine maps for the detection of venous thrombosis in second-generation dual-energy CT (DECT): An ex vivo phantom study. Eur. Radiol..

[14] Bossuyt P.M., Reitsma J.B., Bruns D.E., Gatsonis C.A., Glasziou P.P., Irwig L., Lijmer J.G., Moher D., Rennie D., de Vet H.C. (2015). STARD 2015: An Updated List of Essential Items for Reporting Diagnostic Accuracy Studies. Radiology.

[15] Albrecht M.H., Scholtz J.E., Kraft J., Bauer R.W., Kaup M., Dewes P., Bucher A.M., Burck I., Wagenblast J., Lehnert T. (2015). Assessment of an Advanced Monoenergetic Reconstruction Technique in Dual-Energy Computed Tomography of Head and Neck Cancer. Eur. Radiol..

[16] Altman D.G. (1990). Practical Statistics for Medical Research.

[17] Meisamy S., Bolan P.J., Baker E.H., Pollema M.G., Le C.T., Kelcz F., Lechner M.C., Luikens B.A., Carlson R.A., Brandt K.R. (2005). Adding in vivo quantitative 1 H MR spectroscopy to improve diagnostic accuracy of breast MR imaging: Preliminary results of observer performance study at 4.0 T. Radiology.

[18] Reimer P., Jahnke N., Fiebich M., Schima W., Deckers F., Marx C., Holzknecht N., Saini S. (2000). Hepatic lesion detection and characterization: Value of nonenhanced MR imaging, superparamagnetic iron oxide-enhanced MR imaging, and spiral CT-ROC analysis. Radiology.

[19] Weiss J., Notohamiprodjo M., Bongers M., Schabel C., Mangold S., Nikolaou K., Bamberg F., Othman A.E. (2017). Effect of Noise-Optimized Monoenergetic Postprocessing on Diagnostic Accuracy for Detecting Incidental Pulmonary Embolism in Portal-Venous Phase Dual-Energy Computed Tomography. Investig. Radiol..

[20] Leithner D., Wichmann J.L., Vogl T.J., Trommer J., Martin S.S., Scholtz J.E., Bodelle B., de Cecco C.N., Duguay T., Nance J.W. (2017). Virtual Monoenergetic Imaging and Iodine Perfusion Maps Improve Diagnostic Accuracy of Dual-Energy Computed Tomography Pulmonary Angiography with Suboptimal Contrast Attenuation. Investig. Radiol..

[21] Martin S.S., Pfeifer S., Wichmann J.L., Albrecht M.H., Leithner D., Lenga L., Scholtz J.E., Vogl T.J., Bodelle B. (2017). Noise-optimized virtual monoenergetic dual-energy computed tomography: Optimization of kiloelectron volt settings in patients with gastrointestinal stromal tumors. Abdom. Radiol..

[22] Rossi S., Ghittoni G., Ravetta V., Viera F.T., Rosa L., Serassi M., Scabini M., Vercelli A., Tinelli C., Bello B.D. (2008). Contrast-enhanced ultrasonography and spiral computed tomography in the detection and characterization of portal vein thrombosis complicating hepatocellular carcinoma. Eur. Radiol..

[23] Gines P., Guevara M., Arroyo V., Rodes J. (2003). Hepatorenal syndrome. Lancet.

[24] Salerno F., Gerbes A., Gines P., Wong F., Arroyo V. (2008). Diagnosis, prevention and treatment of hepatorenal syndrome in cirrhosis. Postgrad. Med. J..

[25] Wichmann J.L., Katzberg R.W., Litwin S.E., Zwerner P.L., de Cecco C.N., Vogl T.J., Costello P., Schoepf U.J. (2015). Contrast-Induced Nephropathy. Circulation.

[26] McDonald J.S., McDonald R.J., Williamson E.E., Kallmes D.F. (2017). Is Intravenous Administration of Iodixanol Associated with Increased Risk of Acute Kidney Injury, Dialysis, or Mortality? A Propensity Score-adjusted Study. Radiology.

[27] McDonald R.J., McDonald J.S., Newhouse J.H., Davenport M.S. (2015). Controversies in Contrast Material-induced Acute Kidney Injury: Closing in on the Truth?. Radiology.

[28] Bittner D.O., Arnold M., Klinghammer L., Schuhbaeck A., Hell M.M., Muschiol G., Gauss S., Lell M., Uder M., Hoffmann U. (2016). Contrast volume reduction using third generation dual source computed tomography for the evaluation of patients prior to transcatheter aortic valve implantation. Eur. Radiol..

[29] Nash K., Hafeez A., Hou S. (2002). Hospital-acquired renal insufficiency. Am. J. Kidney Dis..

[30] Rudnick M.R., Goldfarb S., Wexler L., Ludbrook P.A., Murphy M.J., Halpern E.F., Hill J.A., Winniford M., Cohen M.B., VanFossen D.B. (1995). Nephrotoxicity of ionic and nonionic contrast media in 1196 patients: A randomized trial. The Iohexol Cooperative Study. Kidney Int..

[31] Lee H.K., Park S.J., Yi B.H., Yeon E.K., Kim J.H., Hong H.S. (2008). Portal vein thrombosis: CT features. Abdom. Imaging.

